# Novel sensing probe using Terbium-sensitized luminescence and 8-hydroxyquinoline for determination of prucalopride succinate: green assessment with Complex-GAPI and analytical Eco-Scale

**DOI:** 10.1186/s13065-022-00876-0

**Published:** 2022-10-21

**Authors:** Mona S. Elshahed, Safaa S. Toubar, Azza A. Ashour, Rasha Th. El-Eryan

**Affiliations:** Pharmaceutical Analytical Chemistry Department, Faculty of Pharmacy, Helwan University, Cairo, 11795 Egypt

**Keywords:** Prucalopride succinate, Terbium chloride, 8-Hydroxyquinoline, Spectrofluorimetry

## Abstract

A highly sensitive spectrofluorimetric method is developed for the determination of prucalopride succinate (PRU). The method depends on lanthanide-sensitized luminescence due to complex formation between the drug and terbium chloride (Tb^+3^) which is enhanced by the addition of 8 hydroxyquinoline (8HQ) and phosphate buffer (0.02 M, pH 3.2). The calibration curve was constructed over the linear range 10–300 ng/mL after excitation at 226 nm and measuring the emission of the ternary complex at 544 nm. The method was validated according to ICH Q2 (R1) guidelines and showed a good recovery ± RSD of 100.41% ± 1.26, the limits of detection and quantitation were found to be 2.81 and 8.53 ng/mL, respectively. The proposed method was successfully applied for the determination of the drug marketed tablet dosage form and the results were in good agreement with the reference method. Also, the method greenness was evaluated according to Complex-GAPI and analytical Eco-Scale.

## Introduction

Luminescence is derived from a Latin word called (Lumen) which means light, it was firstly used in 1888 by a German physicist. In the process of luminescence; light is emitted from luminescing material as the electrons go back from the excited state to their ground state [1]. The luminescence is divided into fluorescence, which is a temperature-independent process, and phosphorescence, a temperature-dependent process [1]. Lanthanide-sensitized luminescence is considered a chemiluminescent process as the source of energy emitted by terbium is obtained after the formation of a complex between the lanthanide and a chromophore ligand and excitation of that ligand [1].

The lanthanide-sensitized luminescence method is widely used for the determination of organic ligands by forming a stable luminescent complex between the ligand and one of the lanthanide ions; especially terbium (Tb^+3^), erbium (Eu^+3^), and lanthanum (La^+3^) [2]. The luminescence of the formed complex arises from the effective intra-molecularly energy transfer between the overlapped excited triplet state of the ligand to the localized excited state of the lanthanides which is then emitted by a radiative transition; resulting in what is known as antenna phenomena (the energy absorbed by the ligand is emitted through the lanthanides) [3, 4]. That is why the excitation wavelength of the formed complex is specific to the ligand while the emission wavelength is specific to the lanthanides [5]. For terbium-sensitized luminescence; it was found that the cation has a higher affinity toward oxygen-containing compounds or hybrid oxygen–nitrogen compounds [5]. Usually, the formed complex with terbium is characterized by a wide absorption band (due to the energy transfer from the ligand to the lanthanide cation), sharp emission bands located around 490 nm which is corresponding to ^5^D_4_—^7^F_6_ transition, 545 nm which is corresponding to ^5^D_4_—^7^F_5_ transition, 590 nm which is corresponding to ^5^D_4_—^7^F_4_ transition, 620 nm which is corresponding to ^5^D_4_—^7^F_3_ transition and finally at 650 nm which is corresponding to ^5^D_4_—^7^F_2_ transition; the number of formed peaks depend on the nature of the environment [6, 7]. The sharp band that is centered around 545 nm is mostly used for analysis. Also; the formed complex is characterized by large stokes shift and long-life luminescence [8]. Tb^+3^ can’t be satisfied by drug molecules alone as its coordination number is between 6 and 8, binding of Tb^+3^ with water molecules would result in reducing the emitted luminescent light in form of vibrational energy of oxygen in water molecules. Due to this drawback many papers reported the using of secondary ligand forming a ternary complex which intensify the emitted light. For instance; DNA [3], Ethylenediaminetetracetic acid (EDTA) [6], and micellar medium [9] were all used as secondary ligands.

**Fig. 1:**
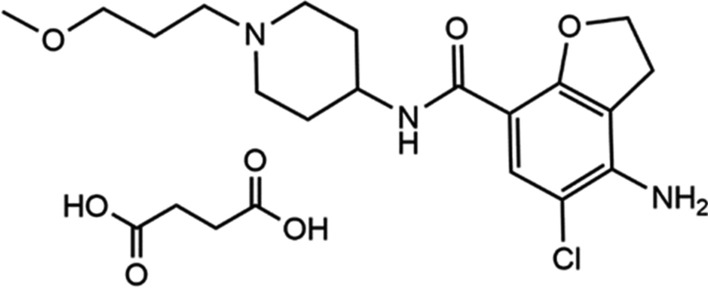
Chemical structure of Prucalopride succinate

Herein, we aimed to use the advantage of the lanthanide-sensitized luminescence to develop and validate an analytical method for the determination of prucalopride succinate (PRU). The IUPAC name of PRU is 4-amino-5-chloro-N-[1-(3-methoxypropyl)piperidin-4-yl]-2,3-dihydro-1-benzofuran-7-carboxamide;butanedioic acid [10] (Fig. 1). The drug is used for the treatment of constipation by stimulating 5-hydroxytryptamine receptors which consequently improves colon motility. The drug was approved to be used in USA for the first time in 2018 after approval from FDA but it was previously approved in Europe in 2009 [11]. By surveying the literature for the developed methods for determination of PRU and to the best of our knowledge, we found few methods for the drug analysis; spectrophotometric methods [12, 13] and chromatographic methods [10, 14–16].

## Experimental

### Apparatus

Jasco FP-6200 Spectrofluorometer was used with xenon lamp (150 W), grating monochromator and 1.0 cm quartz cell. The slit width of the excitation and emission monochromators were adjusted at 10 nm; the spectra were displayed using Spectra Manager FE-6200 Control Driver software, version 1.54.03. Jasco V-630 spectrophotometer, equipped with D2/WI lamp, the spectra were analyzed using Spectra Analysis V-630 Control Driver software, version 2.0804. Hanna pH meter, USA (HI 2211 pH/ORP) with glass electrode. Bench-top sonicator POWERSONIC410; USA.

### Materials and reagents

#### samples

The pure form of PRU was obtained from Marcyrl (Cairo, Egypt) and its purity is certified to be 99.82%. The dosage form Prucasoft® 2 mg prucalopride tablets (batch NO. 2031461) was purchased from the local Egyptian market; each film-coated tablet contains an equivalent amount of 2.64 mg Prucalopride succinate.

#### Reagents

Terbium chloride hexahydrate (Tb^+3^) (its purity 99.9%) and all solvents used (methanol, ethanol, and acetonitrile) of HPLC grade; Sigma-Aldrich, Germany. 8hydroxy-quinoline (8HQ) (its purity 99.5%); BDH, Dubai, UAE. Disodium Ethylenediaminetetracetic acid (disodium-EDTA); Innova Priority Solutions, Cairo, Egypt. Disodium hydrogen phosphate, sodium dihydrogen phosphate, and phosphoric acid, for preparation of phosphate buffer according to European pharmacopoeia [17]; El-Nasr pharmaceutical company, Cairo, Egypt. Hydrochloric acid and ammonium acetate, for preparation of hydrochloric-ammonium acetate buffer according to European pharmacopoeia [17]; Piochem company, Cairo, Egypt. Double-distilled water was used for the preparation of all solutions.

### Preparation of solution

#### Preparation of standard solution

The first standard stock solution of 1 mg/mL PRU was prepared by accurately transferring 25 mg of PRU powder into 25-mL volumetric flask, the dilution was made with water. The second standard stock solution of 0.01 mg/mL was prepared by quantitively transferring 0.1 mL from the first stock solution to 10-mL volumetric flask using a micropipette, the volume was completed to the mark using water. The aqueous standard working solution of 0.001 mg/mL was prepared by quantitively transferring 1 mL from the second standard stock solution to 10-mL volumetric flask and the final dilution was made with water. The standard solutions were kept in the refrigerator and used for 2 weeks.

#### Preparation of tablet solution

To prepare the first tablet stock solution of 1 mg/mL PRU, ten tablets of Prucasoft® (2.64 PRU) were weighted individually then manually crushed in a mortar into a fine powder. An appropriate amount of the fine powder equivalent to 10 mg PRU was transferred into a 10-mL volumetric flask and mixed with an appropriate volume of water. The solution was thoroughly mixed in the sonicator for 15 min, the volume was completed to the mark with water and mixed well then it was filtered through 0.45 µm syringe filter. The second tablet stock solution of 0.01 mg/mL PRU was prepared by quantitively transferring 0.1 mL from the first tablet stock solution to 10-mL volumetric flask using micropipette, the volume was completed to the mark using water. The working tablet solution of 0.001 mg/mL was prepared by quantitively transferring 1 mL from the second tablet stock solution to 10-mLvolumetric flask and the final dilution was made with water.

#### Reagents.

- Aqueous solution of 4 × 10^–4^ M Tb^+3^ was prepared by accurately transferring 0.04 g of Tb^+3^ to 250-mL volumetric flask.
- 4 × 10^–4^ M 8HQ was prepared by accurately transferring 0.04 g of 8HQ to 500-mL volumetric flask, the dilution was made with water. The flask was sonicated for nearly 20 min to ensure dissolving of the reagent.
- 4 × 10^–4^ M EDTA was prepared by accurately transferring 0.03 g of EDTA to 250-mL volumetric flask, the volume was completed to the mark using water.

### General procedure for construction of the calibration curve

Different aliquots from the working standard solution of PRU covering the concentration range 10–300 ng/mL PRU then 0.5 mL of 4 × 10^–4^ M Tb^+3^, 1.5 mL of 4 × 10^–4^ M 8HQ and finally 1 mL of phosphate buffer pH 3.2 were mixed in 10-mLvolumetric flasks. The volume was completed to the mark with water. Each solution was mixed well and allowed to stand for 15 min before measuring. The blank solution was prepared in the same manner without adding the standard solution of the drug. The emission was measured after excitation at λ_ex_ 226 nm and the calibration curve was constructed by plotting the final concentration of the drug against the relative fluorescence intensity (RFI) obtained at λ_em_ 544 nm.

## Results and discussion

### Spectral characteristics

**Fig. 2:**
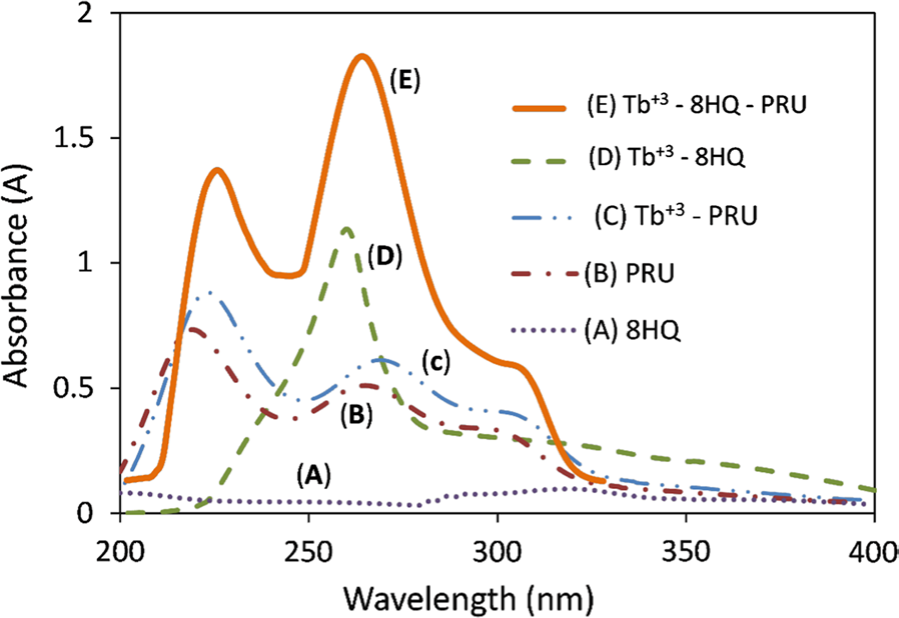
The absorption spectra of PRU, 8HQ, Tb^+3^—8HQ binary complex, Tb^+3^—PRU binary complex and Tb^+3^—8HQ—PRU ternary complex

The absorption spectrum of 5 µg/mL PRU revealed two absorption peaks at 220 and 266 nm (Fig. 2B); which exhibited an enhancement in the intensity and red shift by 3 nm with the addition of 4 × 10^–5^ M Tb^+3^(Fig. 2C). The absorption spectrum of 4 × 10^–5^ M 8HQ revealed no absorption peak (Fig. 2A) which then showed an absorption peak at 260 nm after the addition of 4 × 10^–5^ M Tb^+3^ (Fig. 2D). The absorption spectrum of the ternary complex (Fig. 2E) Tb^+3^—8HQ—PRU showed two peaks at 226 nm and 263 nm with enhancement in the Absorbance (A) which proves the formation of the ternary complex [2].

**Fig. 3:**
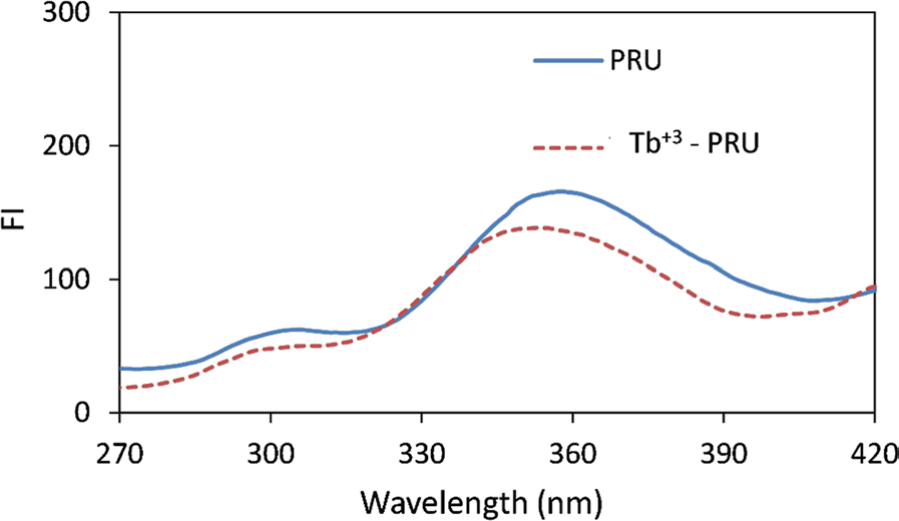
The emission spectra of 300 ng/mL PRU alone and in presence of 4 × 10^–5^ M Tb.^+3^ (λ_ex_ 223 nm)

PRU solution (300 ng/mL) showed a native fluorescent peak in the UV region at λ_em_ 357 nm upon applying λ_ex_ at 220 nm, there is an overlap between the absorption and emission spectra of the drug, as shown in Figs. 2 and 3 which causes an inner filter effect and hinders the use of its native fluorescence as the light emitted from the sample can be reabsorbed again by the sample itself [18]. Also, a possible interference from absorbing species may occur with the native fluorescence of the drug which makes it analytically unuseful in some cases [8].

A solution containing 4 × 10^–5^ M Tb^+3^ and 300 ng/mL PRU was excited at 224 nm, the main three characteristic sharp bands of Tb^+3^ were obtained at 489, 544 and 584 nm. The high stokes shift which could be observed as a large difference between the excitation and emission wavelengths is originated from the nature of Tb^+3^ which has a large gap between the excited state and the ground state [5]. A reduction in the native fluorescence of the drug after addition of Tb^+3^ prove the effective energy transfer from the drug to Tb^+3^ as shown in Fig. 3 [19].

**Fig. 4:**
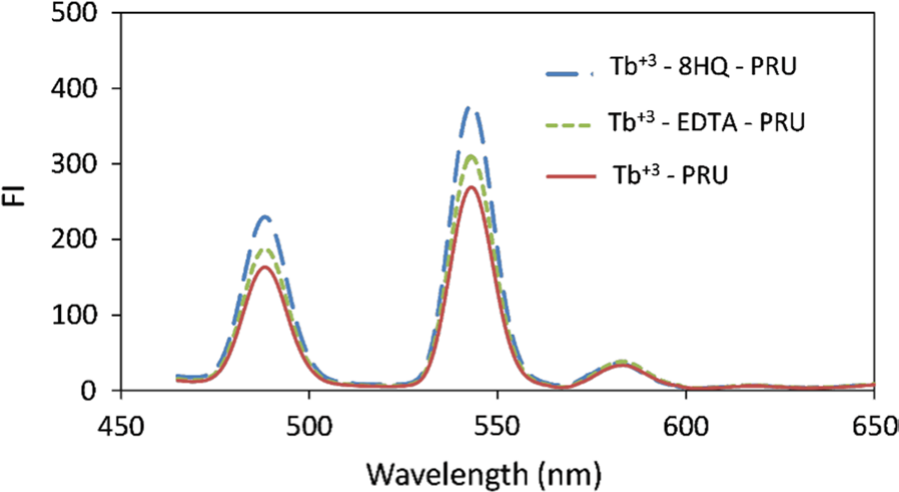
The effect of secondary ligand (4 × 10^–5^ M EDTA or 4 × 10^–5^ 8HQ) on the emission spectra of Tb.^+3^—PRU (λ_ex_ 223 nm)

From the literature, we found that the addition of a secondary ligand that could bind to the Tb^+3^ and displace the solvent molecules, which act as effective quenchers, will intensify the emitted light. EDTA was reported to be used with Tb^+3^ and effectively enhance the luminescence intensity [4]. Also, we found that 8HQ was used previously with La^+3^ for the determination of DNA with the same concept of secondary ligand but to our knowledge, it wasn’t used before with Tb^+3^. Herein we studied both ligands to enhance the luminescence intensity produced from the binary complex of Tb^+3^—PRU. As shown in Fig. 4, the addition of EDTA produced less than 15% enhancement in fluorescence intensity (FI) while the addition of 8HQ intensified the produced luminescence with nearly up to 40%; so, 8HQ was chosen as a secondary ligand in our proposed method.

**Fig. 5:**
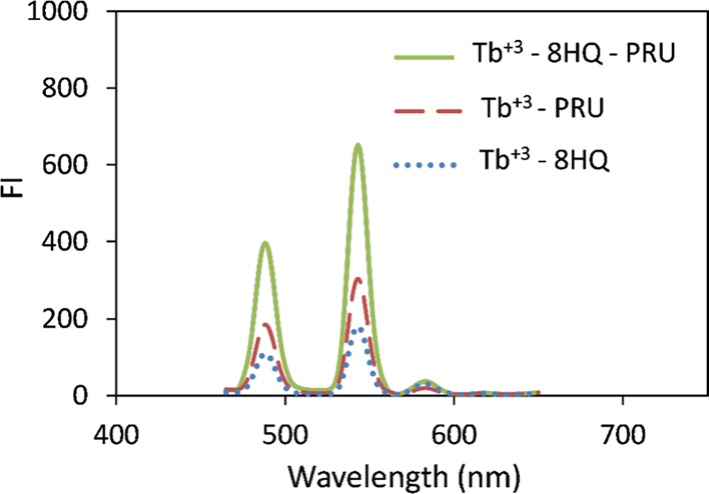
Emission spectra of an aqueous solutions of Tb^+3^—8HQ (2 × 10^–5^ M Tb^+3^ and 6 × 10^–5^ M 8HQ), Tb^+3^—PRU (2 × 10^–5^ M Tb^+3^, 300 ng / mL PRU) and Tb^+3^—8HQ—PRU (2 × 10^–5^ M Tb^+3^, 6 × 10^–5^ M 8HQ and 300 ng / mL Pru using pH 3.2 of phosphate buffer) (λ_ex_ 226 nm)

After optimizing different condition parameters, as it will be shown in section Optimization of general procedure, the fluorescence that was obtained with the ternary complex of Tb^+3^—8HQ—PRU showed higher intensity than that of the binary complex of Tb^+3^—PRU and Tb^+3^—8HQ with nearly 2.5 and 3.5 times enhancement in (FI), respectively, as shown in Fig. 5. The fluorescent intensity that was obtained with the ternary complex showed higher intensity than that of the native fluorescence of the studied drug.

### Optimization of general procedure

All the optimization steps were performed on the ternary complex of Tb^+3^—8HQ—PRU and the binary complex of Tb^+3^—8HQ as a blank.

#### Effect of pH and buffer type

**Fig. 6:**
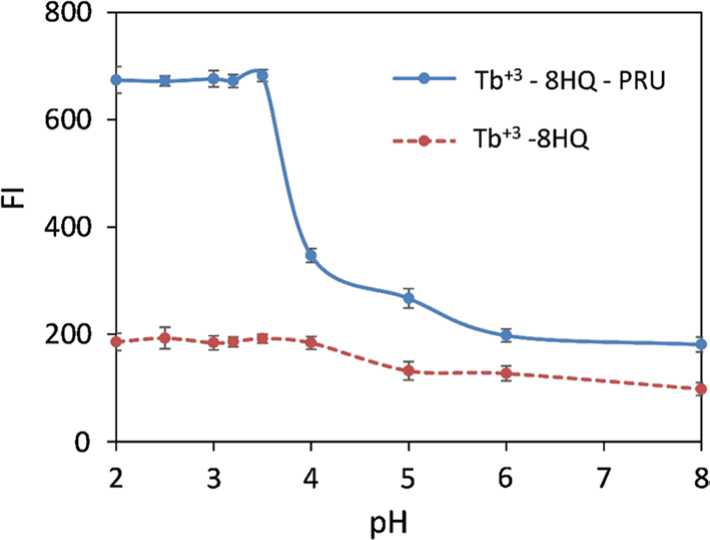
The effect of pH on the fluorescence intensity of Tb^+3^—8HQ (4 × 10^–4^ M Tb^+3^, 4 × 10^–4^ M 8HQ) and Tb^+3^—8HQ—PRU (4 × 10^–4^ M Tb^+3^, 4 × 10^–4^ M 8HQ, 300 ng / mL PRU), using phosphate buffer (0.02 M, pH 3.2) at λ_ex_ /λ_em_ 226/544 nm

Phosphate buffer at 0.02 M was used to study the effect of pH range 2–8. The fluorescence intensity was measured at λ_ex_/λ_em_ = 226/544 nm, the binary complex of Tb^+3^—8HQ was found to be stable over pH range 2–4 while the ternary complex was found to be stable over the pH range 2–3.5 as shown in Fig. 6, pH 3.2 was used for further analysis. Hydrochloric acid- ammonium acetate buffer (0.02 M, pH 3.2) was also investigated and it was found that it gave 29% lower relative fluorescence for the ternary complex than that of phosphate buffer. By using different volumes of phosphate buffer (0.02 M, pH 3.2), no change in the FI was observed so, 1 mL phosphate buffer (0.02 M, pH 3.2) was chosen for further analysis.

#### *Effect of Tb*^+*3*^* concentration*

**Fig. 7:**
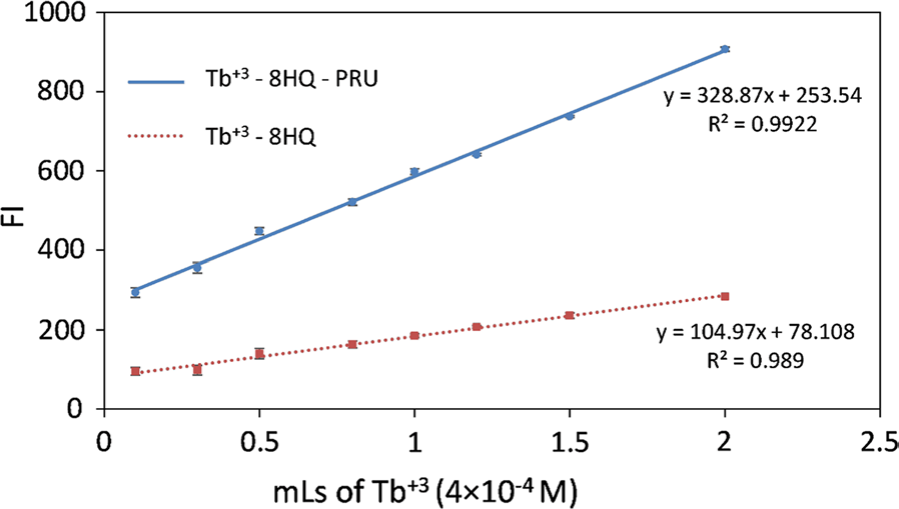
The effect of Tb^+3^ concentration on the fluorescence intensity of Tb^+3^—8HQ (4 × 10^–5^ M 8HQ) and Tb^+3^—8HQ—PRU (4 × 10^–5^ M 8HQ, 300 ng / mL PRU) using 1 mL phosphate buffer (0.02 M, pH 3.2), λ_ex_ /λ_em_ 226/544 nm

The fluorescence of both the ternary complex and the blank was measured with increasing the concentration of Tb^+3^, it was found that the fluorescence intensity for both of them increased quantitively with increasing the terbium concentration (Fig. 7), no plateau was obtained and the same response was reported previously [20]. With trials and errors, 0.5 mL of (4 × 10^–4^ M) Tb^+3^ was. found to be appropriate to obtain a reasonable fluorescence intensity with a final Tb^+3^ concentration of 2 × 10^–5^ M.

#### Effect of 8-HQ concentration

**Fig. 8:**
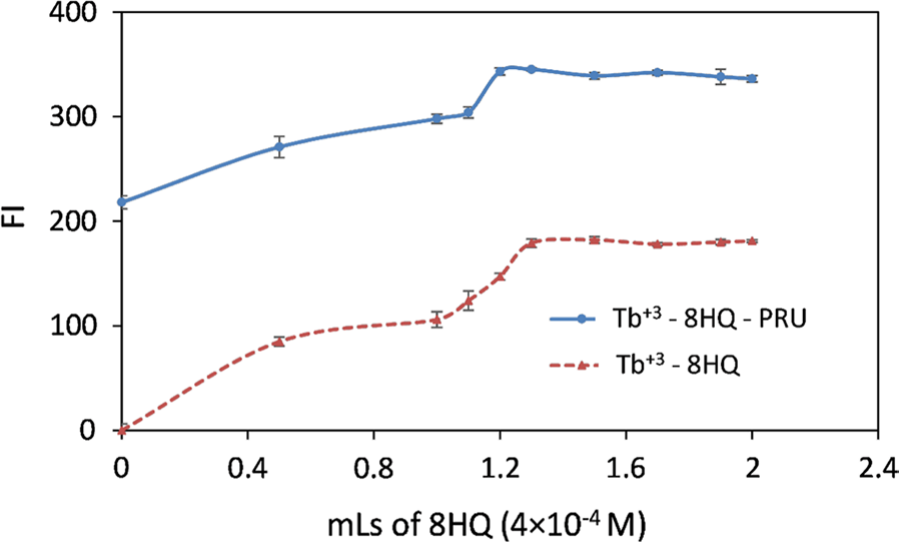
The effect of 8HQ concentration on the fluorescence intensity of Tb^+3^—8HQ and Tb^+3^—8HQ—PRU (2 × 10^–5^ M Tb^+3^, 300 ng / mL PRU using 1 mL phosphate buffer (0.02 M, pH 3.2), λ_ex_ /λ_em_ 226/544 nm

From the Fig. 8, it can be seen that the maximum emission of the binary complex was achieved by adding 1.3 mL from 8HQ solution; while for the ternary complex it was found to be 1.2 mL 8HQ solution. 1.5 mL of 4 × 10^–4^ M 8HQ was used in further analysis to ensure that all Tb^+3^ was bound with the reagent.

#### Effect of diluting solvent

**Fig. 9:**
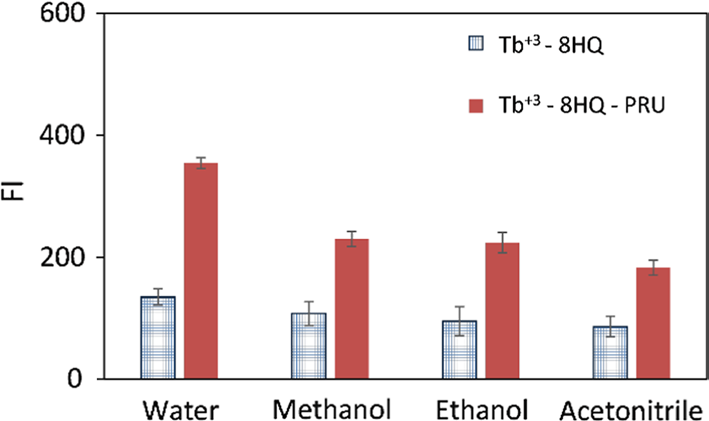
The effect of different diluting solvents on the complex formation of Tb^+3^—8HQ and Tb^+3^—8HQ—PRU (2 × 10^–5^ M Tb^+3^, 6 × 10^–5^ M 8HQ, 300 ng / mL PRU), using 1 mL phosphate buffer (0.02 M, pH 3.2), λ_ex_ /λ_em_ 226/544 nm

Different diluting solvents (methanol, ethanol, acetonitrile) were investigated as shown in Fig. 9. It was found that the organic solvents reduce the intensity of the formed complex with 8HQ, which is in agreement with the previously published method with 8HQ and lanthanum [21]. The fluorescent intensity obtained in water was high and reasonable so it was chosen to be used in subsequent measurements.

#### Effect of addition order

Six different addition orders were investigated as shown in Table 1, it could be seen that maximum FI was obtained when PRU was first mixed with Tb^+3^ then 8HQ was added and finally phosphate buffer.

**Table 1:** The effect of addition order on the fluorescence intensity of Tb^+3^—8HQ and Tb^+3^—8HQ—PRU (2 × 10^–5^ M Tb^+3^, 6 × 10^–5^ M 8HQ, 300 ng / mL PRU)

Addition order	Fluorescence intensity	Standard deviation (SD)
PRU, 8HQ, Tb^+3^, buffer	303	0.72
PRU, Tb^+3^, 8HQ, buffer**	463	0.35
PRU, Tb^+3^, buffer, 8HQ	298	0.29
PRU, 8HQ, buffer, Tb^+3^	336	0.51
Tb^+3^, 8HQ, PRU, buffer	299	0.37
Tb^+3^, 8HQ, buffer, PRU	324	0.39

^**^The selected order of addition

#### Effect of time on complex formation

**Fig. 10:**
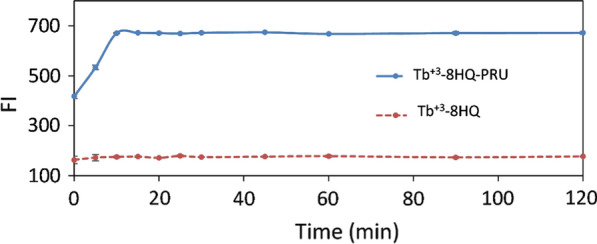
The effect of time on the complex formation of Tb^+3^—8HQ and Tb^+3^—8HQ—PRU (2 × 10^–5^ M Tb^+3^, 6 × 10^–5^ M 8HQ, 300 ng / mL PRU) using 1 mL phosphate buffer (0.02 M, pH 3.2), λ_ex_ /λ_em_ 226/544 nm

The fluorescence intensity was monitored over 2 h. For the ternary complex it was found that the fluorescence intensity was increased gradually up to 15 min and remained constant till 2 h. For the binary complex, it takes 5 min for the complex formation and its fluorescence remained constant for 2 h. All subsequent measurements were taken within time interval ranging from 20 to 30 min (Fig. 10).

#### Effect of UV radiation

To study the stability of the formed complex in the UV radiation, a cuvette containing the ternary complex (300 ng / mL PRU, 2 × 10^–5^ M Tb^+3^, 6 × 10^–5^ M 8HQ and 1 mL phosphate buffer (0.02 M, pH 3.2) were exposed to the UV radiation and the FI has been monitored over 2 h from time 0. The FI almost did not change over the specified time with %RSD 0.21 indicating the complex stability in the UV radiation over the specified time.

### Proposed interaction mechanism of the ternary complex

The binary complex of Tb^+3^—PRU showed the characteristic emission peaks of Tb^+3^ which were enhanced with the addition of the secondary ligand (8HQ) indicating that the energy transfer in the binary complex was incomplete. The observation could be attributed to that; the eight-coordination sites of Tb^+3^ are saturated with oxygen in the drug and in water molecules which cause a non-radiative energy loss through O–H vibration in the solvent molecules, while the addition of 8HQ could bind with the binary complex with electrostatic interaction and replace the water molecules, resulting in the observed enhancement in the emission spectrum [7].

**Fig. 11:**
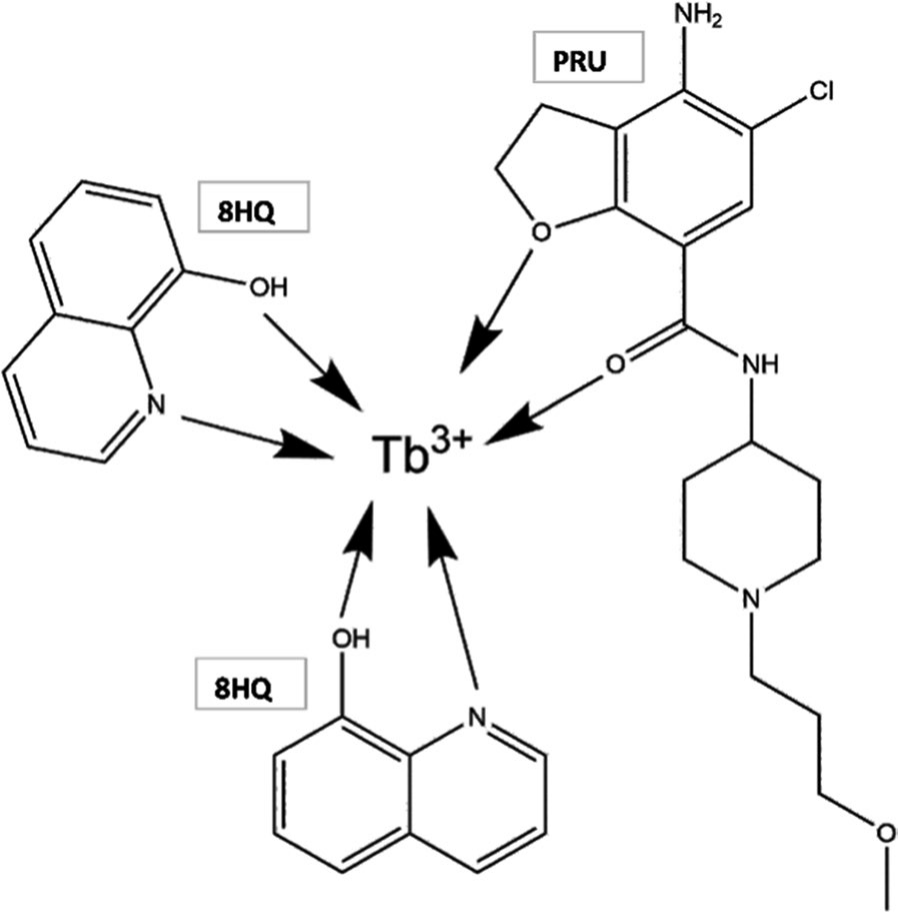
Schematic diagram of the possible structure of the ternary complex Tb^+3^—8HQ—PRU

From the previously studied optimization procedure of Tb^+3^ and 8HQ concentrations under Sects. Effect of Tb^+3^ concentration and Effect of 8-HQ concentration, it could be concluded that 2 × 10^–5^ M Tb^+3^ reacts with 4.8 × 10^–5^ M 8HQ in the ternary complex with ratio nearly 1:2 Tb^+3^:8HQ, the same was obtained in the literature [21]. A proposed mechanism was suggested for the possible structure of the ternary complex as shown in Fig. 11.

### Validation of the analytical method

The proposed method was validated according to ICH Q2 (R1) guidelines [22] regarding the linearity and range, accuracy, precision, limits of detection and quantitation and selectivity.

#### Linearity and range

**Fig. 12:**
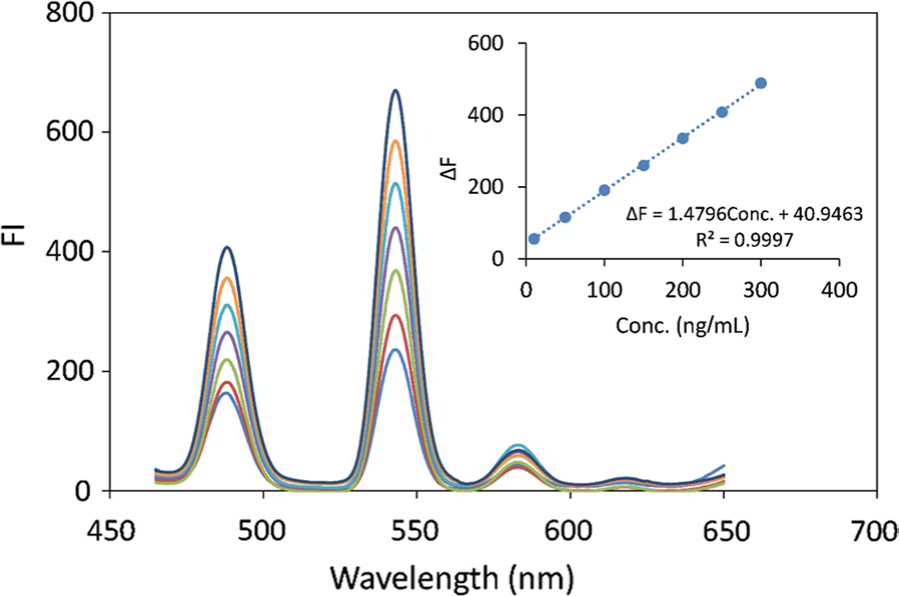
The emission spectra of Tb^+3^—8HQ—PRU ternary complex using 2 × 10^–5^ M Tb^+3^, 6 × 10^–5^ M 8HQ,1 mL phosphate buffer (0.02 M, pH 3.2) and covering the concentration range of 10—300 ng/mL PRU. The inset is the corresponding calibration curve at λ_ex_ /λ_em_ 226/544 nm

After optimizing the condition for the complex formation, different concentrations of PRU were measured as illustrated in section General procedure for construction of the calibration curve and they were found to be linear over the concentration range 10—300 ng/mL with regression equation ΔF = 1.4796Conc. + 40.9463; R^2^ = 0.9997 (Fig. 12); where ΔF is the relative fluorescence intensity obtained by subtracting the fluorescence intensity of the blank from that of ternary complex at different concentrations of PRU. The analytical parameters are shown in Table 2; the high value of the coefficient of determination (R^2^) and small values of residuals standard deviation (S_y/x_) and percent error (%Er) indicate the good linearity of the method.

**Table 2:** Regression data of the linear range for the quantitative determination of PRU in standard solution using the proposed method

Parameters	Standard PRU
Concentration range	10–300 (ng/mL)
Intercept	40.9463
Slope	1.4796
Coefficient of determination (R^2^)	0.9997
% Recovery	100.41
Limit of detection (LOD)	2.81 (ng/mL)
Limit of quantitation (LOQ)	8.53 (ng/mL)
Relative standard deviation (%RSD)	1.3
Standard deviation of residuals (S_y/x_)	6.6
Standard error (S.E)	0.5

Precession data	Repeatability (intra-day precision) *	Intermediate precision (inter-day precision) *
% Mean recovery	99.75	99.67
% RSD	0.36	0.75
% Error (%Er)	0.20	0.44

^*^Average values for three different concentrations, each concentration repeated three times

#### Accuracy

The accuracy of the method was proved by using the standard addition method in which a known concentration from the tablet solution was spiked with three different known concentrations from the standard solution. The results are shown in Table 3, the good recoveries indicate the high accuracy of the proposed method.

**Table 3:** Quantitative determination of PRU in Prucasoft® tablets by the proposed method using the standard addition technique*

Parameters	Conc. Taken (ng /mL)	Conc. Added (ng /mL)	Conc. Found (ng /mL)	%Recovery
Prucasoft® (2 mg/tab)	50	25	75.81	101.08
		50	100.48	100.48
		100	147.34	98.23
% Mean Recovery				100.02
% RSD				1.2

^*^Each result is an average of three determinations

#### Precision

The repeatability and the intermediate precision were evaluated three times per day and on three successive days, respectively; using three different concentrations. The results are shown in Table 2 which prove the good precision of the method indicated by low values of % RSD.

#### Limits of detection and quantitation

From the obtained linearity equation; limits of detection (LOD) and quantitation (LOQ) were calculated using the following equations:

$${\text{LOD }} = { 3}.{3}\sigma /{\text{S}}$$

$${\text{LOQ }} = { 1}0\sigma /{\text{S}}$$

where σ is the standard deviation of response and S is the slope of the regression equation of the calibration curve. LOD and LOQ were calculated and found to be 2.81 and 8.53 ng/mL, respectively (Table 2).

#### Selectivity

The selectivity of the proposed method was proven by its ability to determine the studied drug in pharmaceutical tablet dosage form in presence of other excipients with good % recoveries as shown in Table 4.

**Table 4:** Accuracy and precision data for determination of PRU in tablet dosage form using the proposed method*

Prucasoft ®				Reported method** [10]
Conc. (ng/mL)	50	150	250	% Recovery
% Mean recovery	99.47	99.51	99.29	99.83
% RSD	1.0	0.7	1.6	1.4
% Error (%Er)	0.6	0.4	0.9	0.8
Student’s *t*-test				0.29 (2.78)
F-value				6.61 (19)

^*^Each result is an average of three determinations. **RP-HPLC using C18 column was used, the eluting solvent with the isocratic mood is acetonitrile:0.02 M potassium dihydrogen phosphate in the ratio of 20:80 v/v, the flow rate is 1 mL/min at a λ_ex_ 277 nm

#### Robustness

The method robustness was studied by making a deliberate variation in the method parameters; pH of buffer solution ± 0.1, the volume of phosphate buffer ± 0.02 mL, the volume of 8HQ ± 0.02 mL, and finally the excitation wavelength ± 2 nm. The recovery of the sample was calculated after measuring it under the ideal parameters and with making a minor change through a triplicate analysis, %RSD of recoveries was found to be less than 2% with all studied minor changes.

### Application

The method was successfully applied to determine the studied drug in pharmaceutical tablet dosage form; the recoveries obtained are summarized in Table 4. The proposed method showed good agreement when compared with the reference method [10] regarding accuracy as indicated by the student’s *t*-test and precision as indicated by the F-test [23].

### Assessment of greenness of the method

Green chemistry, also called sustainable chemistry, is aimed to prevent or reduce the negative impact of chemicals on the environment. Developing green analytical methods could be the starting point to change the attitude in paratheatrical industries toward the environment [24]. Many metrics were developed to estimate the greenness of the analytical methods, herein we used both the analytical Eco-Scale and Complex-GAPI assessments.

#### Analytical Eco-Scale

It is one of the very popular metrics, it provides a quantitative assessment of the method greenness, taking into account all the reagents used in the methodology, not only the most hazardous one as with other metrics [25, 26]. The assessment is based on calculating the penalty points which then are subtracted from a base of 100, the obtained value is the Eco-Score of the method. A score of more than 75 reflects an excellent analytical method, a score more than 50 represents an acceptable green method while using of hazardous reagents which increase the penalty point and reduce the score to less than 50 represent inadequate green analysis. As shown in Table 5, the Eco-Score of our method represents an excellent green analysis.

**Table 5:** Analytical Eco-Scale score for the proposed method

	Reagents	Penalty points (PPs)
NaH_2_PO_4_		1
Na2HPO_4_		1
Terbium chloride hexahydrate		1
8HQ		1
		Ʃ4

	Instruments	
Energy		1
Waste		3 + 1
		Ʃ5
Total penalty points	Ʃ9	
Analytical Eco-Scale score	91 (excellent green analysis)	

#### Complementary green analytical procedure index (Complex-GAPI)

**Fig. 13:**
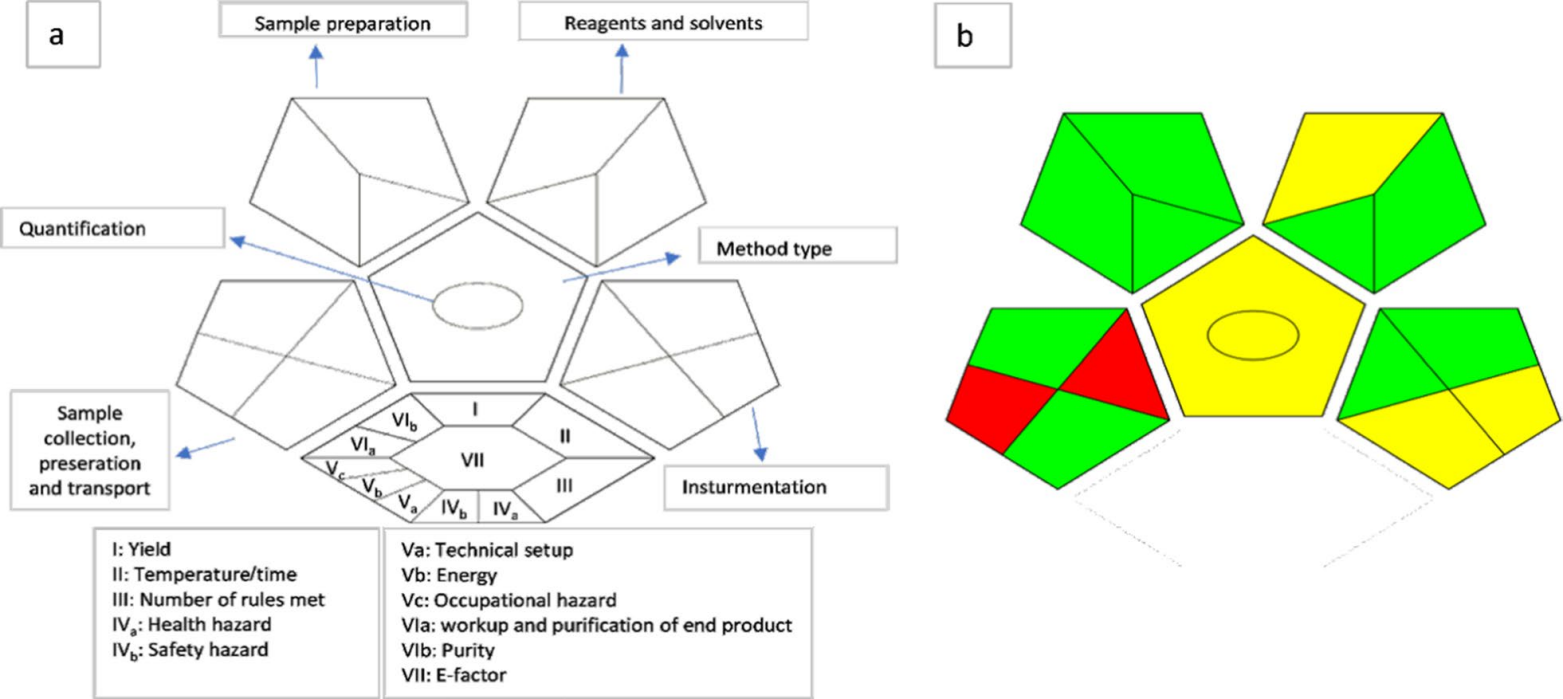
Complex-GAPI assessment of our proposed analytical method

This index is the most recently developed metric to estimate the method greenness. It is an update of the commonly used metric called GAPI [27]. The index provide a widely used metric to evaluate the analytical method greenness [28, 29]. This assessment is represented with five pentagrams which provide a comprehensive evaluation of the entire methodology; starting from sample collection and preparation, solvents and reagents used, the instrumentation, the waste produced and the energy consumption. The additional hexagonal shape at the base of the assessment (Fig. 13a) reflects the pre-analysis steps [30]. This assessment uses a color scale, the red color reflects a high hazardous impact to the environment while green and yellow colors represent a medium and low impact, respectively. For our proposed method (Fig. 13b), the two red regions in the first pentagram of sample collection are mandatory because in the pharmaceutical industry there is a distance between the production center and the quality control department. The hexagonal shape is white as we depend on direct measurement with no pre-analysis preparation. The method showed good greenness with 9 green regions.

### Previous published methods

By comparing our proposed method with the previously published methods for determination of the studied drug as shown in Table 6, our method offers a simple procedure with low consumption of chemicals, no sophisticated steps are required with reasonably high sensitivity compared to the UHPLC-MS–MS that needs very special instruments and use of organic solvents.

**Table 6:** Comparison between our proposed method with previously published methods for determination of PRU

Method	Applications	Linearity range	References
RP-HPLC	Tablets Stability study	2–12 μg/mL	[10]
RP-HPLC	Tablets	50–150 μg/ml	[14]
UHPLC-MS/MS	Rat plasma	0.1–100 ng/mL	[15]
LC-QTOF-MS/MS	Stability study	80–120 μg/mL	[16]
Spectrophotometry	Tablets	5–60 μg/mL	[12]
Spectrophotometry	Tablets	2–10 µg/ml	[13]
Voltammetry	Tablets	0.23–1.06 µg/mL	[31]
Spectrofluorimetric	Tablets	10–300 ng/mL	Our proposed method

## Conclusion

The proposed method provides an effective spectrofluorimetric determination of prucalopride succinate using the ternary complex Tb^+3^—8HQ—PRU. For the first time; 8HQ was as a secondary ligand with Tb^+3^ to form a ternary complex which intensify the emitted fluorescence than that of binary complex of Tb^+3^-PRU. The method was validated according to ICH guidelines and used effectively for the determination of the studied drug in authentic powder with acceptable recovery 100.41 ± 1.26. Also, it was successfully applied to the tablet dosage form. The greenness of the developed method was validated by two matrices, analytical Eco-Scale and Complex-GAPI, our proposed method is proved to be an eco-friendly analytical method.

## Data Availability

The data that support the findings of this study are available from the corresponding author upon reasonable request.
